# The 5′ Spreading of Small RNAs in *Dictyostelium discoideum* Depends on the RNA-Dependent RNA Polymerase RrpC and on the Dicer-Related Nuclease DrnB

**DOI:** 10.1371/journal.pone.0064804

**Published:** 2013-05-20

**Authors:** Stephan Wiegand, Christian Hammann

**Affiliations:** Ribogenetics@Biochemistry Lab, School of Engineering and Science, MOLIFE Research Center, Jacobs University Bremen, Bremen, Germany; University of Dundee, United Kingdom

## Abstract

RNA interference (RNAi) is a gene-regulatory mechanism in eukarya that is based on the presence of double stranded RNA and that can act on both, the transcription or post-transcriptional level. In many species, RNA-dependent RNA polymerases (RdRPs) are required for RNAi. To study the function of the three RdRPs in the amoeba *Dictyostelium discoideum*, we have deleted the encoding genes *rrpA*, *rrpB* and *rrpC* in all possible combinations. In these strains, expression of either antisense or hairpin RNA constructs against the transgene *lacZ* resulted in a 50% reduced β-Galactosidase activity. Northern blots surprisingly revealed unchanged lacZ mRNA levels, indicative of post-transcriptional regulation. Only in *rrpC* knock out strains, low levels of β-gal small interfering RNAs (siRNAs) could be detected in antisense RNA expressing strains. In contrast to this, and at considerably higher levels, all hairpin RNA expressing strains featured β-gal siRNAs. Spreading of the silencing signal to mRNA sequences 5′ of the original hairpin trigger was observed in all strains, except when the *rrpC* gene or that of the dicer-related nuclease DrnB was deleted. Thus, our data indicate that transitivity of an RNA silencing signal exists in *D. discoideum* and that it requires the two enzymes RrpC and DrnB.

## Introduction

Double stranded RNA is the central nucleic acid entity in the gene regulatory mechanism RNA interference (RNAi) [1] that acts in many eukaryotes. Silencing signals are the small, 21–25 nt long double stranded small interfering RNAs (siRNAs), which can lead to transcriptional or post-transcriptional gene silencing (PTGS). Depending on the origin of the siRNA and the level of its double-strandedness, PTGS can manifest in two different mechanisms: mRNA degradation and translational inhibition (summarized in [2]). In most organisms, three core classes of proteins have been identified that are essential for RNAi: RNA-dependent RNA polymerases (RdRPs), Dicer-like proteins and argonaute proteins, next to an ever-growing number of additional auxiliary proteins [3]. The ATP-dependent RNase III Dicer processes double standed RNA in siRNAs [4]–[7]. Argonaute proteins have various functions in RNA silencing [8], and they constitute the catalytic component of the RNA-induced silencing complex (RISC), in which one strand of the siRNAs guides RISC to the sequence homologous mRNA (summarized in [9]). The Slicer activity of the Argonaute cleaves this mRNA in the centre of the double strand formed with the guiding strand [10], [11].

The third class of proteins, RdRPs, can synthesize primer-dependent or -independent complementary RNAs on a single stranded RNA template, and they are required for several mechanisms of RNA-mediated gene regulation, including RNAi [3], [12], [13]. RdRPs frequently serve to amplify an initial silencing signal, as was initially shown for the *C. elegans* RdRP EGO-1 [14]. The siRNAs that trigger the amplification are referred to as primary siRNAs and they can lead to the generation of secondary siRNAs by RdRP activity, which thus amplifies the silencing signal, as summarized in [15]. Individual RdRPs have been shown to have distinct functions in a given organism, as exemplified by the three enzymes of the ciliate *Paramecium tetraurelia* [16].

Because of the directionality of nucleic acids polymerases, secondary siRNA can also be observed 5′ of the original silencing trigger. This 5′ spreading has been observed operational in *C. elegans* in investigations of chimeric *unc-22/gfp* transgenes, from which the term transitive silencing was coined [17]. Recent work has provided evidence for a substantial difference in the effects of primary and secondary siRNAs, since the former can act as triggers but not as templates for activation, while the resulting secondary siRNAs can enforce gene silencing on additional targets without uncontrolled trigger amplification, leading to substantial but fundamentally limited signal amplification [18].

*D. discoideum* encodes three RdRP, named RrpA-C [19]. In a previous study, we have observed that a large fraction of the endogenous siRNAs in *D. discoideum* correspond to the retrotransposon DIRS-1 [20], which was independently confirmed by a recent deep sequencing analysis [21]. At the same time, full-length DIRS-1 transcripts appeared to accumulate in a knock out strains of the *rrpC* gene. Since the genome of *D. discoideum* harbors many copies of this retrotransposon, and siRNAs appear to be covering its entire sequence, it is very difficult to study on this endogenous target, whether transitive silencing [17] exist in *D. discoideum*. To allow for the investigation of this 5′ spreading of a silencing signal, we have established here an artificial system based on the *lacZ* gene, encoding β-D-Galactosidase. We observe post-transcriptional gene silencing upon expression of a hairpin or an antisense RNA in various *rrp* gene deletion strains. Transitivity of the silencing signal is observed in the majority of investigated strains but not when the *rrpC* gene is deleted, or that of the dicer-related nuclease DrnB.

## Materials and Methods

### Growth of amoebae

The *Dictyostelium discoideum* strain A×2 was grown axenically in HL5 medium containing 50 µg/mL ampicillin, 250 ng/mL amphotericin, 10 µg/mL penicillin and 10 µg/mL streptomycin at 22°C in shaking suspension. In strains transformed with pDneo2a *lacZ*, the medium was supplemented with 10 µg/mL geniticin (G418) and for strains transformed with constructs of the pDM326 series, additionally with 10 µg/mL blasticidin.

### Oligonucleotides

DNA oligonucleotides were purchased from Sigma and are listed in Table 1.

**Table 1:** Oligonucleotides used in this study.

Name		Sequence (5′→3′)
Probes for Southern Blots		
#0035	rrpA^Δ^/rrpB^Δ^	GGTGAACAACACAAAGAGAATTG
#0025		GTCGACCAAATAACATTGTAGCGGTTGAAC
#0022	lox^Δ^/rox^Δ^	GCGGCCGCGATTTAAACTATAGACCAAGAATCTTG
#0034		CTCTATATAGATTGATTCTAATTGTTTGG
#0028	rrpC^Δ^	AAGCTTGAGTATCTAAACCATGAAAACTTTAC
#0029		GTCGACCTCTAAAGGTTGTAGATATAAATAAAAAC
#1502	BS^(r)^	CGGGTATATTTGAGTGGAATGAG
#1503		GGATCAATTTAACATTTCTCAACAAG
Probes for the ß-Gal reporter system		
U6		GGATGCCTGCCGGTTGCCCGGAGG
Ddr-6		GGCCAACAATTTTCTCAGCAAGAC
*O120*		CGATCCTTCCCGCCCGGTGCAGTATGAAGG
*O122*		CCGGCGATGAGCGAACGCGTAACGCGAATGGTGCAGCGCG
*O123*		GCAGCAGTTTTTCCAGTTCCGTTTATCCGG
*O130*		GTCACGACGTTGTAAAACGACGGCCAGTGAATCCGTAATCATGG
*O148*		GACACCAGACCAACTGGTAATGGTAGCGACCGGCGCTC
*#1494*	5′upstream	GCCGATCGCGTCACACTACGTC
#1495		CGCGTTACGCGTTCGCTCATCG
*#0190*	ß-gal siRNAs	ACTAGTGCAGAACAACTTTAACGCCGTG
*#0191*		AGATCTCCATGCGGTCGCGTTCG
#0188	siRNAs GFP loop	AACTCGGGCATTCTTGGACACAAATTGGAATAC
*#0189*		TTACTAGTTTCACAGCGTGGCTTCCATCTTCAATGTTGTGTC
Cloning of the ß-Gal reporter system		
#0182	lacZ	CTGCAGAAAAAATGACCATGATTACGGATTCACTG
#0183		CTCGAGTTATTTTTGACACCAGACCAACTGG
#0184	hairpin sense	CACGCTGTGTGTGATCATCTGGTCGCTGG
#0185		ACTAGTCCGTCAGCGCTGGATGC
#0186	hairpin antisense	ACTAGTAAACACGCTGTGCGACCGC
#0187		AGATCTCCGTCAGCGCTGGATGC
#0188	hairpin GFP loop	AACTCGGGCATTCTTGGACACAAATTGGAATAC
#0189		TTACTAGTTTCACAGCGTGGCTTCCATCTTCAATGTTGTGTC
#0190	antisense	ACTAGTGCAGAACAACTTTAACGCCGTG
#0191		AGATCTCCATGCGGTCGCGTTCG
#1196	sense	AGATCTGCAGAACAACTTTAACGCCGTG
#1197		ACTAGTCCATGCGGTCGCGTTCG

### Isolation of genomic DNA

Genomic DNA was isolated as described previously [22].

### PCR conditions

PCR reactions were carried out on 10–100 ng template DNA in 1×PCR buffer (Thermo Scientific), using 0.2 mM dNTPs, 0.5 µM each primer and 1 µL *Taq* polymerase and 2 µL *Pfu* polymerase in a total volume of 50 µL.

### Genes and strains

**Figure 1:**
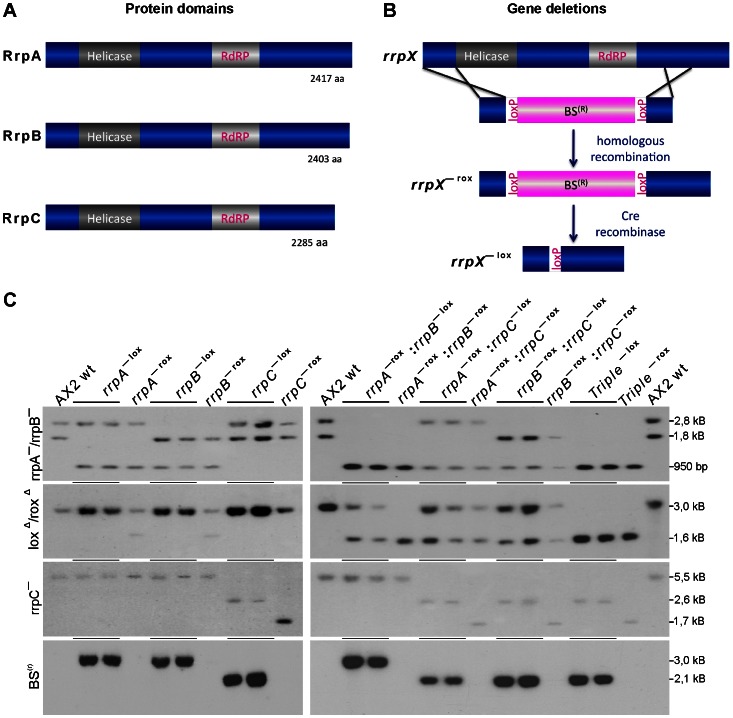
*D. discoideum* RNA-dependent RNA polymerases and gene deletion strains. (A) Protein domain structure of the three RdRPs RrpA, RrpB and RrpC are shown and the sizes of the proteins are indicated. (B) Gene deletions of the *rrp* genes. Shown is for any gene *rrpX*, the design of the deletion construct that will result upon homologous recombination in a disrupted gene lacking any sequence part of the annotated protein domains, yielding the blasticidin resistant (BS^(R)^) strain rrpX¯^lox^. Upon removal of the cassette by transient expression of the CRE recombinase [25], that allows for the generation of multiple gene deletions, rrpX¯^rox^ strain is obtained. The latter strains are used in the analyses presented here. (C) Southern Blot analysis of gene deletion strains. Genomic DNA of A×2 wild type, *rrp* gene single, double and triple deletion strains were digested with the restriction enzymes displayed in Table 2. Upon separation by agarose gel electrophoresis and transfer to a membrane, hybridization was performed using the probes rrpA^Δ^/rrpB^Δ^, lox^Δ^/rox^Δ^, rrpC^Δ^ and BS^(r)^. Expected Signals are listed in Table 2.

The *rrp* genes are listed in the online resource www.dictybase.org with their accession numbers DDB_G0289659 (*rrpA*), DDB_G0291249 (*rrpB*) and DDB_G0280963 (*rrpC*). The latter gene previously had been named *dosA* [19]. All experiments were carried out with A×2, strain 214 and derivatives. The *drnB* ^–^ strain was described recently [21]. Deletion of the *rrp* genes was performed as described using constructs generated by combinatorial cloning [23]. In brief, in each of the used constructs, the two arms for homologous recombination were placed in front of the Helicase domain and after the RdRP domain, respectively to ensure the generation of loss-of-function mutant strains (Figure 1B). Ten µg of linearized vector were transformed into A×2 *D. discoideum* cells by electroporation [24]. Single clone transformants were isolated on *Klebsiella aerogenes* and analyzed initially by PCR and deletions were confirmed by Southern blot (Figure 1), as described recently [23]. Upon excision of the BS^(r)^ cassette by transient expression of the Cre recombinase [25] the gene deletion cassettes could be used again in single knock out strains, yielding the double mutants *rrpA* ^–^:*rrpB* ^–^, *rrpA* ^–^:*rrpC* ^–^ and *rrpB* ^–^:*rrpC* ^–^. Again the BS^(r)^ cassette was excised and the strain *rrpA* ^–^:*rrpB* ^–^ was used for the generation of the *rrpA* ^–^:*rrpB* ^–^:*rrpC* ^–^ (“triple^–^”) knock out strain, from which the resistance cassette was also removed.

### Cloning of the β-Galactosidase reporter system

For the generation of ß-Galactosidase expressing strains, we PCR amplified the coding sequence of the *E. coli lacZ* gene from the plasmid pET-14b lacZ, which was a kind gift of Science Bridge (University of Kassel, Germany), using primers #0182 and #0183. These primers add a *Pst* I site at the 5′ end and an *Xho* I site at the 3′ end for subsequent cloning in pDneo2a. Furthermore, primer #0182 featured an “A run” sequence between the *Pst* I site and the Start codon, which increases expression levels in *D. discoideum* [26]. The PCR product was initially cloned in the vector pJET1.2/blunt (Thermo Scientific) and the resulting construct pJET1.2 *lacZ* was confirmed by sequencing. Using the introduced restriction sites, the A-run modified *lacZ* gene was digested from the cloning vector and cloned in the expression vector pDneo2a [27], which also had been cleaved by *Pst* I and *Xho* I, yielding construct pDneo2a *lacZ*.

For generation of pDM326 ß-gal *sense*, a 985 bp fragment, representing positions 1143–2127 of the *lacZ* gene was PCR amplified from vector pJET1.2 *lacZ*, using primers #1196 and #1197, which add 5′ *Bgl* II or 3′ *Spe* I restriction sites. Upon cloning in the vector pGEM-T easy, the correct insert was confirmed by sequencing. Using the added restriction sites, the insert was excised from the cloning vector and inserted in the vector pDM326 [28], which had also been cleaved with *Bgl* II and *Spe* I. Analogously, pDM326 ß-gal *antisense* was generated, by initially PCR amplifying a 985 bp fragment, representing positions 1143–2127 of the *lacZ* gene. The used primers #0190 and #0191 localize to the same position as #1196 and #1197, however, with a mirrored positioning of the added *Spe* I and *Bgl* II sites. Upon cloning in the vector pGEM-T easy, the correct insert was confirmed by sequencing. Directed cloning in pDM326 resulted in the generation of the antisense construct.

For cloning of the ß–gal *hairpin* construct, initially the sense part of the hairpin, representing positions 1359–1858 of the *lacZ* gene, was PCR amplified from vector pJET1.2 *lacZ* using primers #0184 / #0185 that add *Dra* III and *Spe* I sites to the PCR product at the 5′ end and the 3′ end, respectively. Analogously, an antisense fragment, representing positions 1200–1858 of the *lacZ* gene, was PCR amplified from vector pJET1.2 *lacZ* using primers #0186 / #0187, which add *Spe* I and *Dra* III sites to the 5′ end and a *Bgl* II site to the 3′ end of the PCR product. The sense and antisense PCR products were cloned in the vector pGEM-T easy and confirmed by sequencing. The GFP loop fragment was PCR amplified from vector pDneo2a GFP [27], using primers #0188 and #0189, which add an *Ava* I site to the 5′ end and both, *Dra* III and *Spe* I sites to the 3′ end of the PCR product. To combine these three fragments, initially, the antisense fragment in the pGEM-T easy vector was digested with *Spe* I and *Ava* I, of which the latter targets an endogenous restriction site in that fragment, resulting in the removal of a 162 bp fragment from the 3′ end of the antisense fragment. Using the same restriction enzymes, also the PCR product of the GFP loop was digested and the resulting fragment ligated in the shortened pGEM-T easy ß-gal antisense construct. The resulting construct pGEM-T easy ß-gal antisense-GFP loop was verified by sequencing. From this construct, the insert representing ß-gal antisense-GFP loop was cloned in vector pDM304 [28], using the *Bgl* II and *Spe* I sites in both vectors. Upon verification of the resulting construct pDM304 ß-gal antisense-GFP loop by restriction digest, this vector was digested with *Dra* III and *Spe* I, which allowed for the directed insertion of the sense fragment from pGEM-T easy ß-gal sense by ligation, yielding the construct pDM304 ß-gal hairpin. Finally, by a double digest with *Bgl* II / *Spe* I the ß-gal hairpin construct was inserted in vector pDM326 by directional cloning, resulting in vector pDM326 ß-gal hairpin. Transformation of expression and silencing constructs was carried out by electroporation as described earlier [24]. To exclude insertional artifacts, several independent ß-D-Galactosidase expressing clones were analyzed for stability of enzymatic activity, also upon transformation with an unrelated plasmid that conferred blasticidin resistance to mock the silencing constructs that harbor the same resistance cassette (data not shown).

### RNA isolation

Total RNA was isolated from 5×10^7^ cells of axenically grown *D. discoideum* strains using TRIzol® (Invitrogen) according to the manufacturer's instructions. Finally the isolated total RNA was re-suspended in dH_2_0 and the concentration was determined spectro-photomectrically by using a NanoDrop (Peqlab).

### Northern Blotting

For total RNA blots, 10 µg were separated by gel electrophoresis in a 1,2% GTC-agarose gel in 1×TBE (pH 8.0). Upon capillary transfer to nylon membranes (Roti®-Nylon plus), UV cross-linking was carried out (0,5 Joule/cm^2^). Pre-hybridization and hybridization were carried out in Church buffer (500 mM sodium phosphate (pH 7.2), 1 mM EDTA, 7% w/v SDS, 1% w/v BSA) using DNA oligonucleotides end-labeled with [γ-32P] ATP by T4-PNK (Thermo Scientific) or PCR products that were random-primed-labeled using Klenow Fragment (Thermo Scientific) as described recently [29]. The used oligonucleotides are listed in Table 1. All blots were washed twice for 15 min in 2× SSC, 0.1% w/v SDS, twice for 10 min in 1×SSC, 0.1% w/v SDS and twice for 5 min in 0.5×SSC, 0.1% w/v SDS. For quantitative analyses, blots were quantified against the ethidium bromide stained ribosomal RNA (rRNA) as loading controls.

For small RNA blotting, 20 µg total RNA were separated by gel electrophoresis in a 11% polyacrylamide gel in 20 mM MOPS (pH 7.0). Upon electro-blotting onto a nylon membrane (Amersham Hybond^TM^-NX) for 10 min at 20 V in dH_2_O (semi-dry), RNA was either UV cross-linked as described above or chemically cross-linking as described recently [30]. Small RNAs were probed with 5′ radio-labeled DNA oligonucleotides or random primed probes and the used oligonucleotides are listed in Table 1. All other procedures were carried out as described for Northern Blotting above. As loading controls, U6 RNA was used in UV cross-linked blots and snoRNA DdR-6 for chemical cross-linked RNA, since U6 RNA does not possess a terminal phosphate group that is required for the chemical cross-linking.

### ONPG assay

For the enzymatic analysis of ß-Galactosidase in an o-nitrophenyl-ß-D-galactopyranoside (ONPG) assay [31], 2×10^6^ cells were isolated from an exponentially grown shaking culture, centrifuged for 3 min at 11000 rcf and at 4°C and subsequently washed in 2 mL Soerensen-phosphate buffer (2 mM Na_2_HPO_4_, 15 mM KH_2_PO_4_, pH 6.0). Cells were pelleted again by centrifugation under identical conditions and resuspended in 200 µL ONPG lysis buffer (100 mM sodium phosphate buffer, pH 7.0, 1% w/V NP40) and incubated for 10 min on ice. For the photometric ONPG assay, 5 µL of this cell lysate was mixed with 800 µL Z buffer (100 mM sodium phosphate buffer, pH 7.0, 10 mM KCl, 1 mM MgSO_4_ and 7 mL/L β-mercaptoethanol) and 200 µL ONPG solution (4 mg/mL in 100 mM sodium phosphate buffer, pH 7.0). Absorption measurements were carried out at room temperature at a wavelength of 405 nm. For standardisation, the total protein content in the cell lysate was determined in duplicate by a Bradford Assays [32], using Roti®-Quant (Roth, Karlsruhe, Germany) according to the manufacturer's instructions. For quantitative analysis the ß-Galactosidase activity in the cell lysate was determined according to eq. 1.
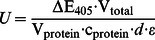
(eq.1)in which U is the ß-Galactosidase activity (in µmol • min^-1^ • mg^-1^), ΔE_405_ the change in absorption (in min^-1^), V_total_ the reaction volume (in mL), V_protein_ the volume of the cell lysate in the reaction (in mL), c_protein_ the protein concentration in the cell lysate (in mg • mL^-1^), d the cuvette path length (in cm) and ε the molar extinction coefficient (4.5 mg • µmol^-1^ • cm^-1^; [33]).

## Results

### Generation of rrp gene deletion strains

The aim of this study is the investigation whether transitivity of a silencing signal exists in *D. discoideum* and if so, whether any of the three RNA dependent RNA polymerases (RdRPs) are involved in this process. The three large proteins are encoded by the genes *rrpA*, *rrpB* and *rrpC*, respectively (Figure 1A) and feature each a helicase domain and an RdRP domain [19]. RrpA and RrpB share 94% sequence identity on the protein level over the entire length, while RrpC is more distinct with 35% identity over 65% of the protein sequence [19]. We generated deletion strains of all three genes using a recently developed one-step protocol [23] that exploits the loxP system for the generation of multiple gene deletion strains [25]. The gene deletion cassettes were designed such that in each gene, the sequences encoding the two domains were completely removed after homologous recombination (Figure 1B). With these gene deletion cassettes, we have initially generated the single knock out strains (*rrpA* ^–^, *rrpB* ^–^ and *rrpC* ^–^, respectively), and subsequently all possible combinations of *rrp* gene deletions. All strains were verified by extensive PCR analysis (data not shown) and Southern blotting (Fig. 1C, Table 2). Since the *rrp* triple knock out strain is viable, the *rrp* genes are not essential in the amoeba. Only the *rrpC* ^–^ strain displayed occasionally irregular (increased) doubling times, which, however, disappeared with time. Thus, none of the gene deletion strains displayed significant defects under axenic growth or during development.

**Table 2:** Expected Signals in Southern Blots of *rrp* gene deletion strains^a^.

Probe		rrpA^Δ^ / rrpB^Δ^		lox^Δ^ / rox^Δ^		rrpC^Δ^	BS^(r)^		
Restriction enzymes		*Sty* I / *Cla* I		*Sty* I / *Cla* I		*Eco* RV	*Sty* I / *Cla* I		
	strain	rrpA	rrpB	rrpA	rrpB	rrpC	rrpA	rrpB	rrpC
Expected signal	wt	1840bp	2779bp	2888bp	2941bp	5537bp	–	–	–
	rrpX¯^lox^	957bp	957bp	3030bp	2991bp	2664bp	3030bp	2991bp	2151bp
	rrpX¯^rox^	957bp	959bp	1630bp	1591bp	1749bp	–	–	–

a rrpX– ^lox^ denotes strains containing the BS(r) casette and rrpX–^rox^ those upon removal of the cassette by transient expression of the CRE recombinase [25].

### A β-D-Galactosidase based reporter system for the analysis of RNA-mediated silencing in *D. discoideum*

To investigate the silencing effect of different RNA constructs, we have generated a heterologous reporter system based on the *Escherichia coli lacZ* gene that encodes β-D-Galactosidase (β-Gal). For the ectopic expression of this transgene in *D. discoideum*, earlier studies have shown that the use of endogenous promoter and terminator sequences results in no undesired obvious changes in the phenotype [31]. At the same time, the enzymatic activity of β-Gal can readily monitored quantitatively in ONPG assays [34]. Initially, the A×2 wild type and all *rrp* gene deletion strains as well as a strain, in which the *drnB* gene is disrupted [21] were transformed with the expression vector pDneo2a lacZ and grown under geniticin selection. All resulting strains expressed β-Gal as monitored by ONPG assays (data not shown).

**Figure 2:**
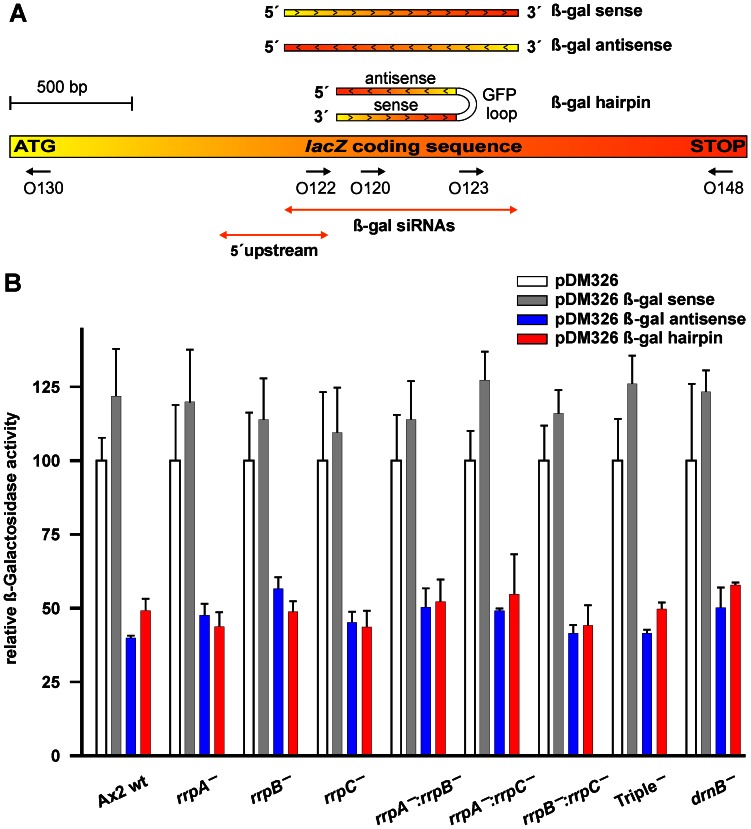
The β-Galactosidase reporter system. (A) The *lacZ* coding sequence is shown in the centre with the 5′end in yellow and the 3′end in orange. On top, the sense and antisense RNAs (each 985 nt long) and the hairpin RNA with 500 bp and a 136 nt long GFP loop are shown. The same color is used as for the *lacZ* gene. These three RNAs are positioned with respect to the relevant sequence in the *lacZ* gene. Black double-headed arrows are shown for oligonucleotide probes and orange double-headed arrows for random primed probes. All probes are positioned relative to the *lacZ* coding sequence. The size marker holds for all constructs shown. (B) Relative β-Galactosidase activity determined in ONPG assays. For the indicated strains, average values from three independent measurements are shown, and bars indicate the standard deviation. For each strain, measurements of cells transformed with the empty pDM326 vector (white) are set to 100%. Values for pDM326 β-gal sense (grey), pDM326 β-gal antisense (blue) and pDM326 β-gal hairpin (red) are shown.

To induce PTGS, these strains were subsequently transformed with a second vector encoding for one of two different RNA silencing constructs, either an antisense RNA or a hairpin RNA directed against lacZ mRNA (Fig. 2A). Both the 985 nt long antisense RNA and the hairpin RNA were placed centrally in the ORF of the lacZ mRNA. The hairpin RNA features 500 bp that are joined by a long loop of 136 nucleotides. As sequence for the loop nucleotides, we chose a partial GFP sequence, which is otherwise not present in the wild type amoeba. We anticipated that the extended size of the loop would allow us to follow its fate upon processing of the hairpin double strand.

The silencing constructs were cloned in the pDM326 vector [28], which is not expected to insert chromosomally. Thus, the transformants were grown under constant blasiticidin selection. To exclude any side effect on β-Gal activity due the presence of this antibiotic, all ONPG measurements were carried out relative to a strain transformed with an empty pDM326 vector. As further control we expressed a partial β-gal sense RNA, positioned identically to the used antisense RNA (Fig. 2A).

### Post-transcriptional silencing by antisense and hairpin RNA

**Figure 3:**
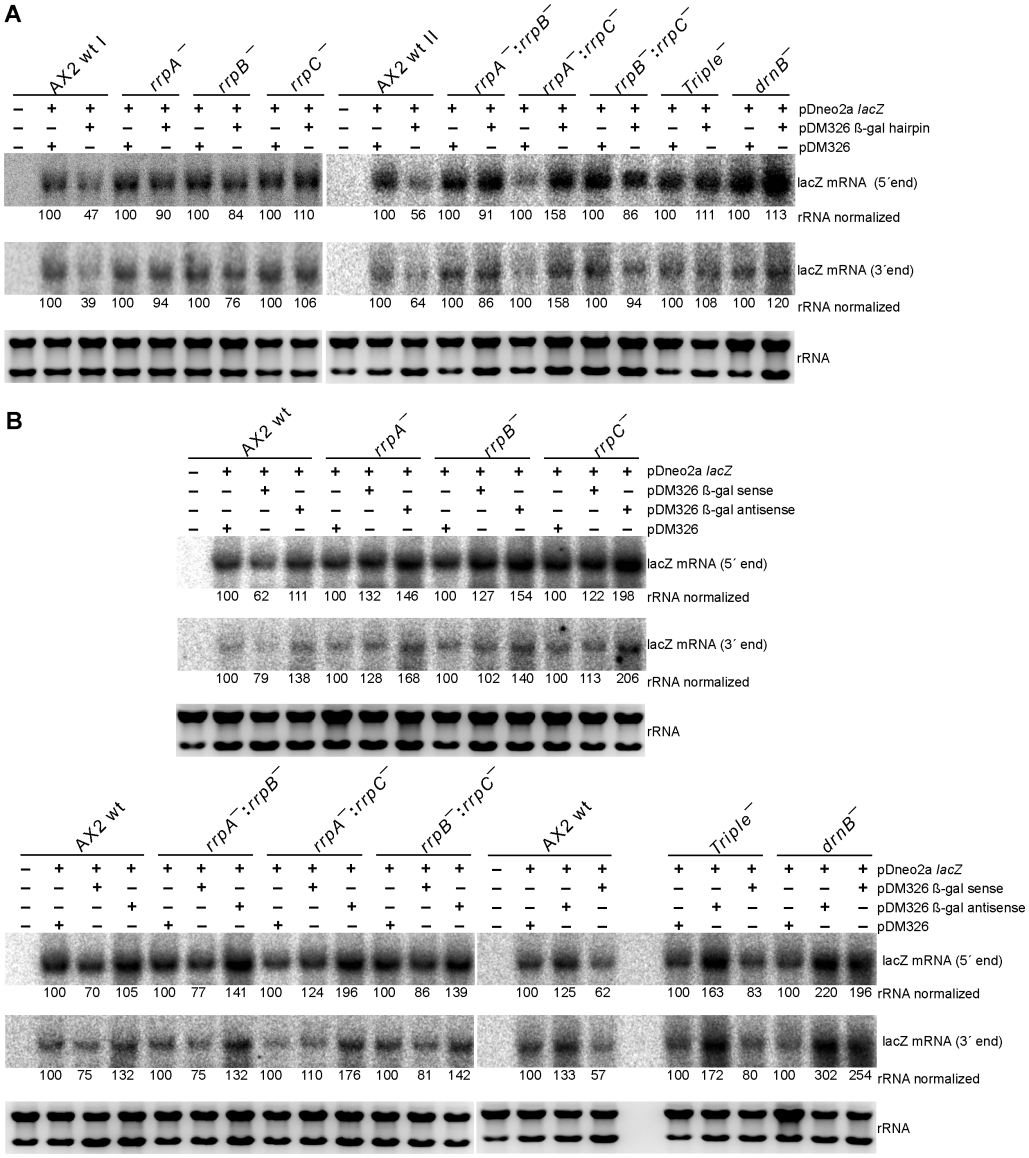
Northern Blots of lacZ mRNA. Transformants of (A) pDM326 β-gal hairpin or (B) pDM326 β-gal sense and pDM326 β-gal hairpin antisense in the indicated strains were analyzed using probes O130 (top) and O148 (middle), which bind to the 5′ end and the 3′ end of the mRNA, respectively. Ethidium bromide stains of the ribosomal RNAs (bottom) serve as loading controls. After normalization to rRNA amounts, lacZ mRNA levels are set to 100% for each strain transformed with the empty pDM326. The values for (A) pDM326 β-gal hairpin or (B) pDM326 β-gal sense and pDM326 β-gal hairpin antisense refers to that background signal.

To investigate the silencing potential of the β-gal hairpin RNA, we transformed A×2 wild type cells, drnB^—^ and the seven *rrp* gene deletion strains with pDneo2a lacZ and either the β-gal hairpin RNA expressing vector or the empty pDM326 vector. The ONPG assays revealed a uniform reduction of the enzymatic activity of β-Gal by approximately 50% in either strain (Fig. 2B). We presumed this observation be due to a processing of the β-gal hairpin RNA to siRNAs and subsequent mRNA degradation by RNAi. To test this, we carried out a Northern Blot analysis on *lacZ* mRNA, using the two probes O130 and O148 (Fig. 2A). This analysis revealed that the mRNA levels varied; to our surprise, however, the observed variation was not dependent on the presence of the β-gal hairpin RNA (Fig. 3A). For example, *lacZ* mRNA was apparently less stable in the rrpA^—^/rrpC^—^ double knock out strain when transformed with the empty pDM326 vector than with the pDM326 β-gal hairpin construct. Independent Northern blots revealed an intrinsic low stability of the *lacZ* mRNA in *D. discoideum* (data not shown), that did not appear to be additionally increased by the presence of the β-gal hairpin RNA.

In analogy to the experiments carried out for the hairpin RNA constructs, also the transformants of the sense and antisense RNA expressing constructs were analyzed. We observe in ONPG assays no significant influence by the sense RNA expressing construct. The antisense RNA, however, led to a reduction in the enzymatic activity of β-Gal similar to that seen for the hairpin RNA (Fig. 2B). Again, the lacZ mRNA stability did not appear changed by the presence of the antisense constructs (Fig. 3B). The antisense RNA itself was refractory to visualization in Northern Blots using the probes O122 and O123 (data not shown), indicating fast turnover of this RNA, which still gave rise to the reduction in enzymatic activity (Fig. 2B). In summary, this data indicated that the reduced β-Gal activity seen upon transformation with either hairpin or antisense construct was not due to lacZ mRNA degradation.

### Generation of siRNAs in the presence of RNA silencing constructs

**Figure 4:**
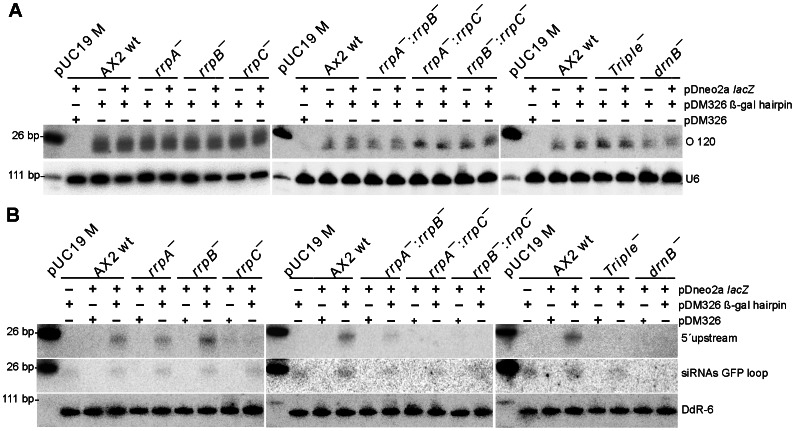
Northern Blot Analysis of siRNAs in the β-gal hairpin expressing strains. (A) Small RNAs in the indicated backgrounds, using oligonucleotide probe O120. (B) Analysis of siRNAs 5′ upstream of the original silencing signal (top) using probe 5′upstream and siRNAs derived of the GFP loop (bottom) in the indicated strains. Loading and transfer efficiency is monitored by probing for U6 RNA (A) or Ddr-6 RNA (B). pUC19 M denotes pUC 19 DNA/*Msp* I Marker (#SM0221 Fermentas).

**Figure 5:**
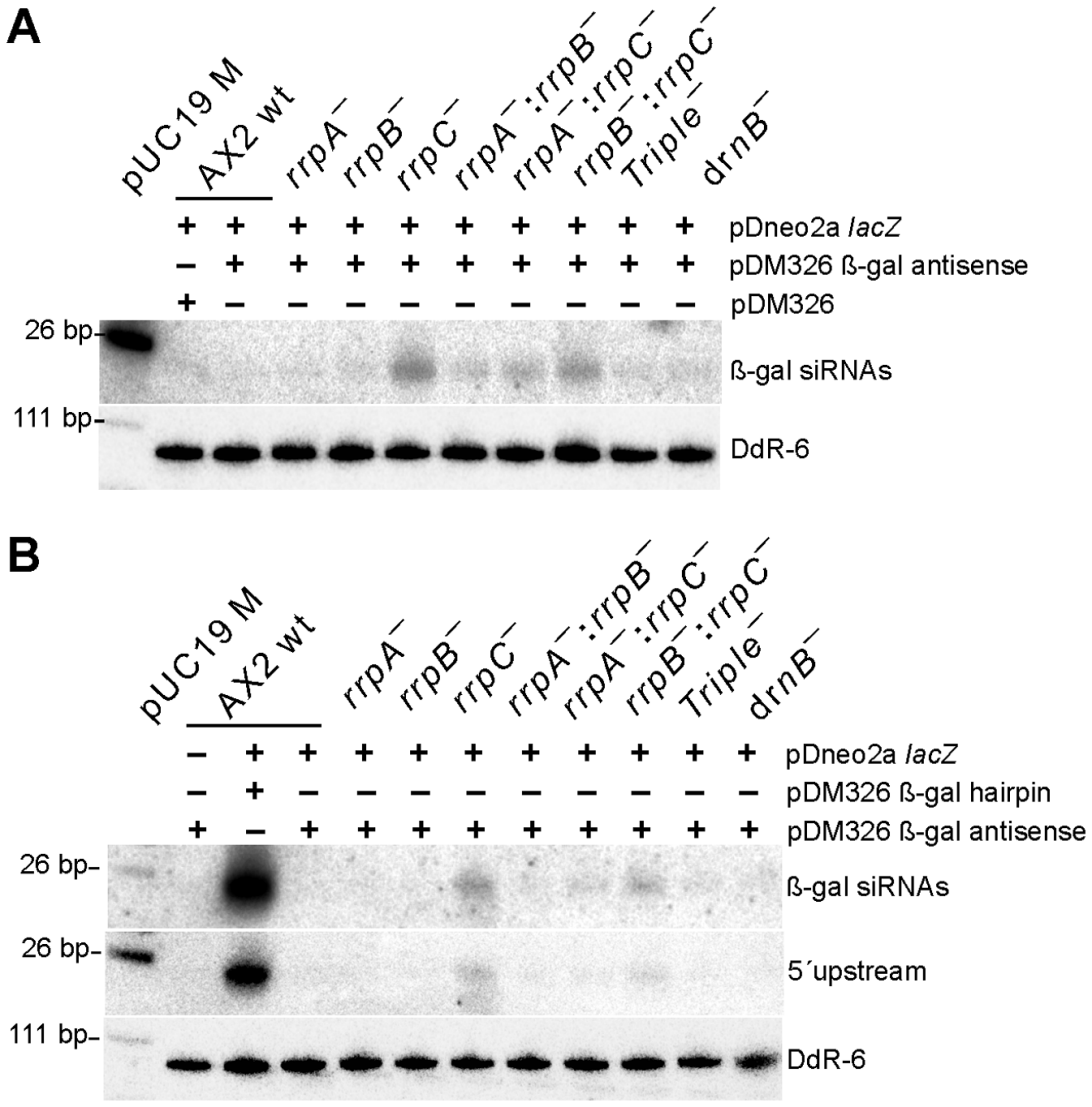
Northern Blot Analysis of siRNAs in the β-gal antisense expressing strains. (A) Small RNAs in the indicated backgrounds, using the random primed probe β-gal siRNAs. (B) Comparison of small RNA levels in the indicated strains expressing β-gal antisense or β-gal hairpin, using probe β-gal siRNAs (top) or probe 5′upstream (bottom). Loading and transfer efficiency is monitored by probing for Ddr-6 RNA. pUC19 M denotes pUC 19 DNA/*Msp* I Marker (#SM0221 Fermentas).

Since the presence of neither the β-gal hairpin RNA nor the β-gal antisense RNA had apparently an effect on *lacZ* mRNA stability, we analyzed next whether siRNAs could be observed at all in the presence of the silencing constructs. For this purpose, we employed β-gal siRNA probes (Fig. 2A) for detection in Northern Blots. A uniform pattern of siRNAs was observed in all strains transformed with the pDM326 β-gal hairpin construct, independent of whether or not the target *lacZ* mRNA was present (Fig. 4A). As expected, the empty pDM326 vector that served as negative control did not show any siRNAs. The siRNAs appeared as broad bands and occasionally, this signal split up into two distinct bands, as can be seen for example for the A×2 wild type in the blot in the middle of Fig. 4A. Different to this, siRNAs in strains transformed with the antisense construct could only be observed in the rrpC and the rrpC double knock out strains (Fig. 5A). The level of siRNAs in these strains was considerably lower than that observed in a strain with the β-gal hairpin RNA construct, as inferred from a comparison of the signal strengths (Fig. 5B).

### Observation of RNA silencing signal transitivity

To investigate whether transitivity can be observed in the β-Gal system introduced here, we have located the β-gal hairpin RNA centrally on the *lacZ* mRNA, which should allow us to monitor secondary siRNAs, in case they do appear (Fig. 2A). Since all nucleic acid polymerases catalyze their synthesis reactions by reading the template 3′→5′, such secondary RNAs are expected 5′ of the original silencing trigger with respect to the template sequence. We again employed Northern blotting, however, this time we used a probe that ends 24 nt in front of the β-gal hairpin (siRNA 5′upstream, Fig. 2A). Our analysis revealed the appearance of secondary siRNAs in A×2 wild type and the *rrpA* and *rrpB* gene deletion strains, but not in either strain, in which the *rrpC* or *drnB* genes were deleted (Fig. 4B). From this data we infer that 5′ spreading of this RNA silencing signal occurs in *D. discoideum* and that for the hairpin RNA, RrpC and DrnB are involved in transitivity. While we observed antisense RNA derived siRNAs using the probes β-gal siRNA and 5′ upstream (Fig. 5) that both overlap (at least partially) with the antisense RNA (Fig. 2A), our attempts to visualize transitivity of the silencing signal in case of the antisense RNA failed, when we employed a probe that would bind putative siRNAs exclusively 5′ of the antisense RNA construct (data not shown).

In the hairpin construct, we had cloned a GFP-derived loop sequence. To analyze the fate of this loop sequence, we re-probed this blot (Fig. 4B, top) with a GFP specific probe and identified siRNAs in all investigated strains with the exception of the *drnB* deletion strain (Fig. 4B, middle). From this data we infer that the loop sequence of the used construct gives rise to siRNAs and that DrnB is involved in their production.

## Discussion

The social amoeba *D. discoideum* encodes three RdRPs that have been shown earlier to be involved in the silencing of endogenous retrotransposons [20]. In this study, we have used an artificial reporter system based on β-D-Galactosidase, whose expression we aimed to silence post-transcriptionally by the use of either a hairpin or an antisense RNA (Fig. 2A). A colorimetric assay revealed a similar level of reduction of the enzymatic activity for both types of silencing constructs (Fig. 2B). The steady state level of the lacZ mRNA, however, appeared not to be affected by the presence of either silencing construct (Fig. 3). For the hairpin construct, a uniform level of siRNAs was observed in all investigated strains (Fig. 4) and it therefore appears likely that the reduction in enzymatic activity might be due to a translational inhibition, rather than to mRNA degradation. Normally, the perfect complementarity of the hairpin-derived siRNAs to the lacZ mRNA would be expected to trigger mRNA degradation [2]. Our data point towards a similar phenomenon as in the plant kingdom, where perfectly base-complementary small RNAs also appear to trigger frequently translational inhibition rather than mRNA degradation, as recently summarized [35]. In a previous report from the Nellen lab, a similar approach was used to silence β-D-Galactosidase by means of a hairpin construct [19]. The construct used in that study consisted of a large double stranded RNA that covered the first 800 bp of the lacZ mRNA and the formed hairpin had the relative order sense-loop-antisense strands. That construct triggered a full RNAi response as shown by the absence of lacZ mRNA (and thus of β-D-Galactosidase enzymatic activity), co-current with the accumulation of β-gal siRNAs. This RNAi response was dependent on the RdRP RrpA. Unlike shown by us, the appearance of the β-gal siRNAs was strictly dependent on the presence of the lacZ mRNA in that study, indicating that the hairpin construct was not processed alone. The hairpin construct used in our current study resulted in a non-degradative response, that in principle might be attributable to the three main differences in the design of the two molecules: their position with respect to the target RNA, the sizes and the relative orientation of the sense and antisense proportion of the hairpin. The 50% reduction in β-D-Galactosidase that we report here in all cases, was also observed when a short 100 bp hairpin construct was used with the strand order employed by the Nellen lab [19]. This indicates that the non-degradative RNA-mediated inhibition, that we report here, can be triggered independent of the strand order in the silencing constructs. Also, the difference in the sizes of the hairpin loops, 500 bps vs. 800 bps seems unlikely to cause the observed differences, although they cannot be formally be ruled out. It seems thus most attractive to attribute the differences to a positional effect of the two constructs. The RrpA-dependent full RNAi response thus would require that the hairpin covers the Start codon, which does not permit to study whether or not transitivity of an RNA silencing signal occurs in *D. discoideum*. Although we did not trigger full RNAi with the positioning of our hairpin RNA construct, we could show that this 5′ spreading of small RNAs indeed exists in the amoeba, as monitored by the appearance of secondary siRNAs that cannot be derived of the original trigger (Figs. 2A and 5B). In their generation, the two enzymes RrpC and DrnB appear to be involved, since these secondary siRNAs disappear in knock out strains of the encoding genes (Fig. 5B). If the secondary siRNAs were directly transcribed as 21mers by RrpC, one would not expect an influence by DrnB. It thus appears tempting to speculate that these secondary siRNAs might be processing products of DrnB that uses RrpC-synthesized longer double-strands as substrates. An association of RdRPs with Dicer proteins has been described before [36], [37], and additional proteins might be required for this association, as exemplified by the Tudor domain protein Eri-5 in *C. elegans* [38].

The siRNAs that are derived of the GFP loop appear to be independent of any of the *rrp* gene products, but they depend on DrnB. Formally, the GFP loop is single-stranded. An inspection of predicted secondary structures of this sequence by the Mfold program [39], however, revealed a considerable degree of (imperfect) base pairing (data not shown). DrnB has been shown earlier to be the enzyme responsible for the processing of the *D. discoideum* microRNA precursors that also feature imperfect base complementarity [21], [40]. Thus, the appearance of the GFP siRNAs might be attributable to the secondary structure of the original loop sequence.

In terms of enzymatic activity of β-Galactosidase, the β-gal antisense construct seemed to have the same effect than the β-gal hairpin construct (Fig. 2). Our analysis of the siRNAs, however, revealed significant differences between the two types of silencing constructs. The post-transcriptional inhibition that the antisense RNA construct causes in all investigated strains (Fig. 2B) might not depend on the presence of siRNA, which are observed only in a sub-set of strains (Fig. 5A). Thus, it appears possible, that the antisense RNA directly interacts with the β-gal mRNA, thereby causing the observed translational inhibition. The antisense RNA construct appears to give rise to siRNAs only in deletion strains of the *rrpC* gene, alone or in combination with either of the other two *rrp* genes, but, surprisingly, not in the triple knock out strain. An enrichment of small RNAs in a deletion strain of an RdRP has also been reported for example for the *Arabidopsis thaliana* enzyme RDR2 [41].

Why should siRNAs accumulate in a strain that lacks an enzyme that is supposed to synthesize RNA? One explanation might be that the responsible RrpC acts in an RNA-primer dependent fashion, a mode of action that has been shown to be operational in the *A. thaliana* RdRP Rrp6 [42] and in the *Neurospora crassa* enzyme QDE-1 [43]. In this model, the observed siRNAs in the *rrpC* gene deletion strains would be unused *substrates* rather than products. This, however, does not explain why they disappear in the triple knock out strain. One explanation for this latter observation could be that RrpA and RrpB be responsible for the siRNAs that are seen in the antisense RNA expressing *rrpC* gene deletion strain (Fig. 5A), similar to the recently reported specialization of RdRPs in *P. tetraurelia* [16]. If that was the case, it might serve to explain the disappearance of antisense RNA derived siRNAs in the triple *rrp* deletion strain. Alternatively, this latter observation might indicate some kind of compensatory mechanism in that triple knock out strain.

In summary, our data indicate distinct, but partially overlapping activities of RdRPs in the generation of siRNAs in *D. discoideum* in strains transformed with antisense and hairpin RNA silencing constructs. We report only for the hairpin RNA, but not for the antisense RNA silencing construct, the appearance of a transitive silencing signal that localizes 5′ to the original trigger. These transitive RNAs appear, however, dispensable for the post-transcriptional silencing effect, for which the presence of the original trigger might suffice.
